# Integrating Milk Metabolite Profile Information for the Prediction of Traditional Milk Traits Based on SNP Information for Holstein Cows

**DOI:** 10.1371/journal.pone.0070256

**Published:** 2013-08-21

**Authors:** Nina Melzer, Dörte Wittenburg, Dirk Repsilber

**Affiliations:** Institute for Genetics and Biometry, Leibniz Institute for Farm Animal Biology, Dummerstorf, Mecklenburg-Western Pomerania, Germany; CNR, Italy

## Abstract

In this study the benefit of metabolome level analysis for the prediction of genetic value of three traditional milk traits was investigated. Our proposed approach consists of three steps: First, milk metabolite profiles are used to predict three traditional milk traits of 1,305 Holstein cows. Two regression methods, both enabling variable selection, are applied to identify important milk metabolites in this step. Second, the prediction of these important milk metabolite from single nucleotide polymorphisms (SNPs) enables the detection of SNPs with significant genetic effects. Finally, these SNPs are used to predict milk traits. The observed precision of predicted genetic values was compared to the results observed for the classical genotype-phenotype prediction using all SNPs or a reduced SNP subset (reduced classical approach). To enable a comparison between SNP subsets, a special invariable evaluation design was implemented. SNPs close to or within known quantitative trait loci (QTL) were determined. This enabled us to determine if detected important SNP subsets were enriched in these regions. The results show that our approach can lead to genetic value prediction, but requires less than 1% of the total amount of (40,317) SNPs., significantly more important SNPs in known QTL regions were detected using our approach compared to the reduced classical approach. Concluding, our approach allows a deeper insight into the associations between the different levels of the genotype-phenotype map (genotype-metabolome, metabolome-phenotype, genotype-phenotype).

## Introduction

In dairy cattle, traditional milk traits are recorded regularly during the standard milk performance test to monitor e.g., the status of health of the cows and the feeding in order to apply this information for breeding purposes. In general, milk traits for monitoring the state of health are not sufficiently sensitive regarding diagnostic efficiency, e.g., acetone is an accepted indicator for ketosis [1], but is increased only if ketosis is acute. Hence, in the last years an increased trend to find new molecular traits, such as metabolite which can be used as helpful indicators, has been observed. These new molecular traits are expected to improve several applications in this field, e.g., to allow for the possibility to detect diseases earlier or to find new opportunities for non-invasive techniques to monitor metabolic processes in cows. Such enhancements also play an important role for the economic aspect. In the recent literature, new molecular traits were investigated and proposed to be used as indicators. For example, Klein *et al.* [2] proposed the ratio of milk glycerolphosphocholine to phosphocholine as an indicator for the risk of ketosis. The study of Cabrita *et al.* [3] suggested that heptandecanoic acid (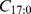) has the potential to be an indicator for protein deficiency. Farr *et al.* [4] proposed to use lactic acid for the detection of mastitis in its early stage. A possible strategy to reveal metabolites important for the prediction of traits of interest, in the following termed as important metabolites, is to apply statistical learning methods which allow variable selection [5] to obtain a measure of importance for each metabolite (as proposed by Melzer *et al.* [6]). In our case, investigated traditional milk traits were considered surrogates for interesting health or management traits. Milk metabolites or groups of metabolites revealed to be important could serve as possible biomarker or biosignature candidates [6]. In general, an important metabolite can be considered a new molecular milk trait, and genetic effects on it may be analyzed with prediction methods from the field of genomic selection [7], [8]. In a recent study, milk metabolites were considered new molecular traits and their genetic variability was investigated [9]. Here, we are interested to find single SNPs with a genetic impact on the important metabolites. Estimated SNP effects may be used to predict genetic values in future generations (test generations). Today it is common to genotype only elite animals, mostly bulls, because of the cost of a high-density panel e.g., Illumina® SNP Chip 50K. Hence, it is also of interest to design low-density SNP panels (3K–6K), based on SNPs selected from the high density SNP panel, which can be used for a broader screening. A low density SNP panel should cover as many milk traits associated with breeding (selection) goals as possible in order to obtain an appropriate prediction precision for several traits [10]. To determine an appropriate SNP subset from a high-density SNP panel, different strategies were proposed in the recent literature. For instance, Habier *et al.* [11] proposed to use equally spaced SNPs to obtain a SNP subset for several traits. Weigel *et al.* [12] used Bayesian Lasso to find an optimal SNP subset for one trait, in which SNPs were ranked based on their genetic effects. A similar study is presented by Moser *et al.* [13] who used ridge regression and partial least squares regression to find an appropriate SNP subset for several production traits.

In this context, we that prediction precision may increase if SNP subsets determined for a molecular species, in our case metabolites, with an impact on the trait of interest, in our case milk traits, are used for prediction. It is possible that important SNPs, which have little genetic effect on a milk trait, may have stronger genetic effects on the corresponding important metabolites. We expect that SNPs with a strong genetic effect on a milk trait (e.g., the SNP in the region of DGAT1 [14], [15] has a large impact on fat) show also a strong genetic effect on at least one of the determined important metabolites.

To address these presumptions, our metabolite approach employs different sub-steps, in which the metabolome level was considered for genetic value prediction in addition to the SNP information. The following prediction precisions were compared: metabolite SNPs, all SNPs (classical approach), important SNPs determined for a milk trait (reduced classical approach), only SNPs which were within or close to known QTL regions (QTL approach) A special evaluation design was applied, i.e., an invariable double 10-fold cross-validation design, enabling direct and fair comparisons between the different approaches. Our main focus was to compare the prediction precisions of the different approaches A second objective was to compare positions of selected SNPs with known QTL positions (enrichment analysis).

## Materials and Methods

### Experimental Data

Our experimental data set includes traditional milk traits and SNP genotypes (Illumina® Bovine SNP 50K) of 1,305 Friesian Holstein cows reared on 18 farms in Mecklenburg-Western Pomerania. The traditional milk traits were measured via infrared spectroscopy (Kombi-FOSS, FT6000-FC, FOSS, Hillerød, Denmark) during the standard milk performance test at the State Control Association for Quality Inspection (LKV, Güstrow, Germany). One milk sample per cow was taken between the 21st and 120th day of the first lactation. Further, the Max Planck Institute of Molecular Plant Physiology (Potsdam-Golm, Germany) measured milk metabolite spectra, which were obtained via GC-MS ([16]) of the hydrophilic phase of milk. For this purpose, a specific experimental design was implemented as balanced as possible to enable an unbiased correction and analysis regarding the following factors: GC-MS batch (day of milk measurement), half-sib structure (sire effect), farm and test day [17]. More detailed information of the preparation of the metabolite profiles can be found in [6]. In total, 187 known metabolites were identified, and three unknown metabolites were measured.

Each cow had less than 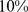 missing SNP genotypes. SNP data were subjected to further quality checks. SNPs with unknown position according to the annotated bovine genome (Btau4.2, [18]) were deleted. Further, SNPs were excluded if MAF was 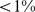, if the Hardy-Weinberg equilibrium was not fulfilled (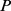-value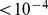, [19]), or if a SNP locus had more than 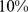 missing values over all cows. In our experimental data set, 40,317 SNPs met all criteria. The rarely missing SNP genotypes were imputed using Beagle v3.2 [20].

The narrow-sense heritability (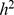) was estimated using a sire model (R package nlme [21], [22]) for each milk trait. This analysis reveals that fat content (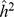 = 0.23), protein content (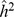 = 0.24) and pH value (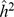 = 0.39) show the highest estimated narrow-sense heritability and therefore they were chosen for the presented analyses. It is known that the number of animals and heritability of a trait have an important influence on the accuracy of the prediction of genetic values (e.g., [23], [24]). In a previous study [6], relationships within these milk metabolites and milk traits as well as relations between milk metabolites and milk traits were deeper investigated using univariate as well as multivariate analysis methods. The Pearson correlation coefficients between all milk metabolites and chosen milk traits were in a range of [−0.14; 0.19] for fat content, and [−0.28; 0.34] for protein content and [−0.18; 0.12] for pH value [6].

### Known QTL regions for fat content and protein content

For the implementation of the QTL approach, the cattle QTL database (cattleQTLdb; [25]) was searched to determine known QTL regions of the bovine genome based on the given cattleQTLdb markers for fat and protein. Entries of the cattleQTLdb were filtered for: trait milk fat percentage and milk protein percentage, analysis type equal to QTL, breed equal to Holstein, and chromosome number, flanking markers (of the confidence interval of the QTL) or peak markers had to be specified. The location of selected cattleQTLdb markers is given in the genetic unit centiMorgan (cM). Then, these markers were assigned to Btau4.2 (as used for the experimental data), using the corresponding marker information from the National Center for Biotechnology Information (NCBI; [26]), to obtain marker positions in the physical unit base pair (bp). In total, 34 QTL regions were associated with fat, and 50 QTL regions with protein. The QTL marker positions used for both milk traits are listed in Table S1. Additionally, the known quantitative trait nucleotide (QTN) DGAT1 [15] was considered a QTL for fat and protein. The position of another known QTN for protein, ABCG2 [27], was already covered by a QTL. Based on the filtered QTL marker positions (bp) it was possible to select SNPs which are close to a QTL peak marker or between two flanking markers of a QTL region:

- QTL region: all SNPs between left (end bp) and right (start bp) flanking marker of the QTL interval.
- QTL peak: left and right SNP next to the peak position.
- DGAT1: a SNP directly in the DGAT1 region (DGAT1: chromosome 14, position 411,147–446,810 bp, www.ncbi.nlm.nih.gov accessed 2011 February 18). The SNP is located on 443,937 bp and termed DGAT1-SNP.

The joint set of SNPs (a)–(c) were termed *QTL-SNPs* in the following analyses.

### Cross-validation scheme

**Figure 1:**
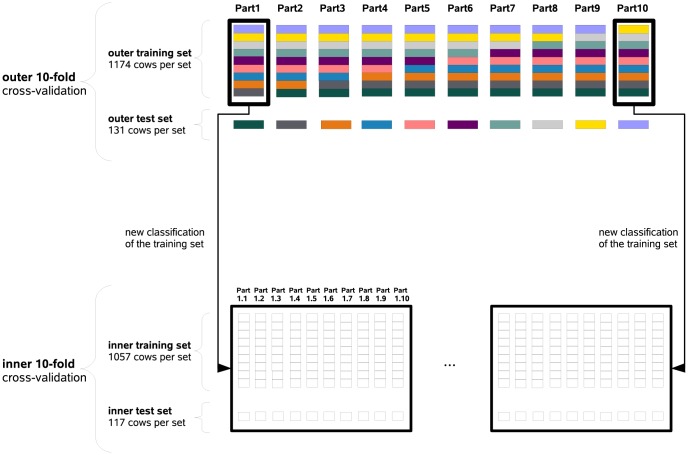
Scheme of the invariable double 10-fold cross-validation (CV) design. To obtain the outer 10-fold CV, which represents a classical CV [5], the whole data set is first divided into ten equal parts considering the half-sib structure, which results in the 10 outer test sets. The outer training set for each test set is created by merging the remaining nine test sets. The outer cross-validation is only used for the genetic value prediction. To enable optimization, i.e., in our case to find optimal milk metabolites for a milk trait, an inner CV is necessary. The inner 10-fold CV is created based on the 10 outer training sets, where each outer training set is again divided in 10 equal parts.)

In general, a cross-validation design is necessary, since we did not have separate experimental data as test set available. Thus, to enable investigations on the experimental data set, it was first divided to obtain a classical 10-fold cross-validation, which is termed outer cross-validation [5].The whole data set was divided into 10 equal parts with equal proportions of half-sib families, representing the outer test sets (cf. Figure 1). To create a corresponding outer training set for a test set, the remaining outer test sets were merged. In detail, to create training set No. 1 for test set No. 1, the following test sets were combined: test set No. 2 (Part2) to test set No. 10 (Part10). This was realized for each test set and thus each cow appeared exactly once in each outer test set. This 10-fold cross-validation is used only for the final prediction of genetic values (cf. Section “Analysis design”).

To enable optimization of subsets, i.e., metabolites or SNP subsets (cf. ection Analysis design), a further inner 10-fold cross-validation is necessary. The inner 10-fold cross-validation was obtained by dividing each outer training set into 10 equal parts representing the inner test sets, and the corresponding inner training sets were assembled as explained above for the outer training sets.

The invariability of the design and the use of the same seeds for the random number generator in the analysis ensures the comparability of the different approaches.

### Analysis design

The following three-step analysis design was performed to investigate associations between three levels of data: SNP genotypes, standardized metabolites and milk traits.

#### Step 1 - inner 10-fold cross-validation

The following statistical model [6] was fitted to metabolites and milk traits (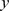):
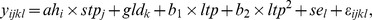
with
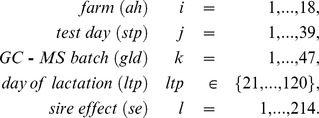
The interaction 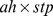 (63 levels) of *farm* and *test day*, *GC-MS batch*, and linear and quadratic regression on *day of lactation* with regression coefficients 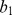 and 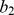 were considered fixed effects. The *sire effect* 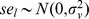 and the residual effect 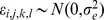 were considered as random. *Sire effect* accounts for the half-sib structure, and sires were assumed to be unrelated. Based on the pedigree data received from the computing center (vit Verden, Germany), cows were assigned to 192 sires, but 22 animals had unknown sires treated as if offspring from independent sires. Note that in the case of milk traits, the factor *GC-MS batch* was excluded from the linear model.

The standardized residuals of metabolites and milk traits were used for the regression of milk traits on metabolite profiles with random forest (RF, [28]; R package randomForest [29]) and partial least squares (PLS, [30]; R package mixOmics [31]). Both regression methods enable variable selection, which allows the extraction of the importance of each metabolite for the prediction of the investigated milk traits. For this purpose, the mean decrease in accuracy for RF and the vip function measure [31] for PLS were used. A metabolite was defined as important for a specific milk trait if its measure of importance was larger than the 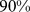 quantile of all metabolite importances in each inner cross-validation run and for each regression method. In this step, the prediction precision (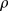) was defined as the correlation between estimated and observed milk trait values. Analyses were implemented in R [22].

#### Step 2 - outer training set

The impact of each SNP on either important metabolites or milk traits was estimated. An SVS method similar to Ishwaran & Rao [32], [33] was applied, including the estimation of the systematic effects as in the linear model and additive genetic effects covered by SNPs. SVS was run using Gibbs sampling with the following settings: 100,000 iterations were used, the first 40,000 of which were disregarded as burn-in phase. Three chains were produced for each trait, and the mean values of estimated genetic effects were determined. These mean values were used in combination with the proportion of non-zero genetic effects to determine significant genetic effects, representing the important SNPs, using an empirical selection method (as described in [33]). SVS was implemented in Fortran. The different important SNPs observed for each of the important metabolites for a milk trait were combined and termed *metabolite SNPs*. The set of important SNPs for an investigated milk trait directly found by SVS was termed *reduced SNPs*.

After this step was completed, the important SNPs were rated related to the known QTL. For this, we used the over-representation analysis which is a type of enrichment analysis [34]. The aim of this analysis is to determine if a list of genes, representing the gene set, is over-represented (more genes than expected by chance) with regard to another gene list, representing the target set. A specific reference set is applied to quantify how likely the over-representation is, which is calculated following the hypergeometric distribution [35]. In our case, the entirety of SNPs represent the reference set. The target set corresponds to the QTL-SNPs, and we investigated its enrichment with regard to the SNP subsets detected in the metabolite approach and the reduced classical approach. The analysis was performed in R, using the function phyper to calculate the *P*-values based on the hypergeometric distribution, the significance level 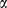 was set to 0.05.

#### Step 3 - outer 10-fold cross-validation

Different SNP subsets were used to estimate genetic effects on milk traits using SVS (same settings as in Step 2): (a) metabolite SNPs, (b) reduced SNPs, (c) all SNPs, (d) QTL-SNPs. Here, mean values of estimated genetic effects in the outer training set were used to predict the genetic values in the outer test set. In this step, the prediction precision (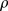) was defined as the correlation between the predicted genetic values and the observed characteristics of a milk trait.

Finally, the observed prediction precisions of the different approaches were rated. The observed prediction precision for the reduced classical approach and the metabolite approach were assessed regarding the obtained average SNP subset. For the rating, a Wilcoxon signed-rank test for paired samples was applied to determine if the observed prediction precisions differed significantly among the various investigated SNP subsets for SVS (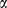 was set to 0.05). This analysis was realized in R.

To evaluate the reduced classical approach and the metabolite approach, it was tested if SNP subsets determined for a milk trait were superior to random subsets. To quantify the significance of the observed prediction ability for the original design, we applied a resampling approach for which SNP subsets were chosen randomly for each investigated milk trait. For each of the 10 outer cross-validation runs 100 SNP subsets were drawn at random corresponding to the observed quantity of SNPs in the respective approach and Step 3 was processed. Thus, the evaluation was based on resamplings, resulting in an empirical distribution of prediction precisions (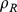). It was counted how often a 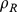 value was larger or equal than the observed prediction precision (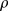). The significance level 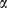 was set to 0.05.

This step was also used to investigate the prediction ability using all SNPs or only SNPs selected by SVS (from Step 2) for each important milk metabolite. Finally, the prediction precisions of the two different SNP subsets were rated using the Wilcoxon signed-rank test for paired samples (see above).

## Results

### Predicting milk traits from metabolite profiles

Two regression methods (RF and PLS) were applied to determine important metabolites for the investigated milk traits. The observed mean prediction precisions were similar for both methods, e.g., 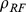 = 0.63 and 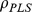 = 0.64 for protein. Protein showed the highest mean prediction precision, whereas mean prediction precisions for fat and pH value were about 0.35 for both regression methods. The important metabolites and their occurrence counts over the 10 inner cross-validation sets are listed in Table 1. In this table, the Pearson correlation coefficients between important milk metabolites and milk traits are listed (adapted from [6]). It is obvious that mostly milk metabolites have an impact as they show a strong correlation to the investigated milk trait. An exception represents threonic acid detected by pH value which has not a direct biological association with the milk trait, but in multivariate analysis it has strong correlation to other revealed milk metabolites related to the investigated milk trait (cf. Figure S1). In Figure S1 the correlations within important milk traits for each investigated milk trait are presented.

**Table 1:** Information about the important metabolites detected within inner 10-fold cross-validation (CV) runs.

Milk trait	metabolite	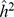	P	Counts in 10-CV	No. of SNPs	*ρ_all_*	*ρ_subset_*	*P*-value
Fat content	1,3-Dihydroxyaceton	0.09	0.19	10	5.30	0.06	0.03	0.38
	Arabitol	0.21	0.19	10	16.90	0.14	0.11	0.23
	Aspartic acid	0.17	−0.13	10	29.00	0.14	0.06	0.03*
	Butanoic acid, 4-amino-	0.18	−0.09	1	4.00	0.13	−0.04	n.a.
	Galactitol	0.00	−0.14	10	18.10	−0.01	−0.08	0.06
	Glucaric acid-1,4-lactone	0.05	−0.12	10	7.40	0.05	−0.05	0.03*
	Muramic acid, N-acetyl-	0.04	0.12	1	6.00	−0.03	−0.02	n.a.
	myo-Inositol-1-phosphate	0.18	0.13	8	6.88	0.12	0.03	0.01*
	Pyroglutamic acid	0.15	−0.12	10	41.50	0.15	0.06	0.01*
	Pyruvic acid	0.08	0.11	4	10.75	0.05	−0.02	0.25
	Sedoheptulose, 2,7-anhydro-, beta	0.00	−0.10	1	4.00	−0.02	0.03	n.a.
pH value	Alanine, beta-	0.22	−0.18	8	10.00	0.14	0.09	0.08
	Arabitol	0.21	0.11	3	18.00	0.17	0.11	0.25
	Glutaric acid, 2-hydroxy-	0.48	0.11	4	33.25	0.38	0.32	0.12
	Glycerol-2-phosphate	0.11	−0.15	10	25.60	0.14	0.14	1
	Glycerol-3-phosphate	0.18	−0.13	7	53.57	0.22	0.19	0.58
	Glycine	0.21	−0.18	10	20.60	0.15	0.08	0.03*
	Phenylalanine	0.03	−0.13	1	8.00	−0.02	0.13	n.a.
	Threonic acid	0.11	0.00	1	4.00	−0.01	0.04	n.a.
	Tryptophan	0.05	−0.11	1	10.00	0.14	0.08	n.a.
	Tyrosine	0.01	−0.13	1	15.00	0.14	0.17	n.a.
Protein content	2-Piperidinecarboxylic acid	0.37	−0.21	10	23.50	0.17	0.09	0.03*
	Adipic acid, 2-amino-	0.19	−0.28	10	24.60	0.16	0.06	0.00*
	Alanine	0.16	−0.13	3	9.00	0.05	0.04	1
	Arabitol	0.21	0.34	10	16.30	0.14	0.09	0.08
	Asparagine	0.06	−0.23	10	12.30	0.12	0.02	0.01*
	Aspartic acid	0.17	−0.22	10	28.40	0.14	0.05	0.01*
	Butanoic acid, 2-amino-	0.27	−0.24	10	29.10	0.14	0.07	0.05*
	Cinnamic acid, 3,4,5-trimethoxy-, trans-	0.02	0.30	10	4.90	0.06	0.04	0.56
	Glyceric acid-3-phosphate	0.09	0.23	4	23.50	0.10	0.08	0.88
	Glycerol-2-phosphate	0.11	0.19	1	29.00	0.06	0.13	n.a.
	Glycerol-3-phosphate	0.18	0.30	10	55.10	0.22	0.17	0.11
	myo-Inositol-1-phosphate	0.18	0.27	10	6.50	0.12	0.07	0.19
	Phosphoenolpyruvic acid	0.41	0.25	7	36.29	0.22	0.17	0.11
	Pyroglutamic acid	0.15	−0.18	10	41.80	0.15	0.10	0.03*
	Spermidine	0.02	0.28	10	11.20	0.07	0.02	0.28
	Thiazole, 4-methyl-5-hydroxyethyl-	0.20	0.21	7	11.43	0.05	0.04	0.69

The table presents the number of occurrences in 10 CV runs (Counts in 10-CV) as well as the average number of selected SNPs (No. of SNPs) over the corresponding CV runs for each important metabolite. In addition, results of the genetic value prediction using all SNPs (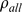) and selected SNPs (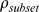) for each metabolite are presented, using the outer 10-fold cross-validation runs. Moreover the 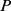-values (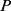-value) obtained from the Wilcoxon signed rank test are listed. The test was applied when a milk metabolite was detected in more than two inner cross-validation runs (i.e., Counts in 10-CV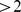). Additionally, the estimated narrow-sense heritabilities (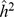) as well as the Pearson correlation coefficients (P; adapted from [6]) obtained between each milk trait and important milk metabolite are presented. For the latter the whole data set was used.

* - significant difference (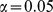).

For fat content 11 different metabolites were found to be important, e.g., 1–3-dihydroxyaceton, aspartic acid and galactitol, six of which were found in each inner cross-validation run. In total, 10 different important metabolites were found for pH value, of which only glycerol-2-phosphate and glycine were found to be in each inner cross-validation run. For protein, 16 metabolites, e.g., alanine, asparagine and pyroglutamic acid, were detected as important, and 11 of them were observed in all inner cross-validation runs. Arabitol, aspartic acid and pyroglutamic acid were important for both fat and protein and they were observed in all inner cross-validation runs.

### Determining important SNPs via SVS

The average number of SNPs selected by SVS for each important milk metabolite is listed in Table 1. For the metabolite approach (the reduced classical approach) the average number of SNPs selected by SVS is: 129 (30) SNPs for fat content, 302 (88) SNPs for protein content and 114 (80) SNPs for pH value. In general, the average number of important SNPs was larger for the metabolite approach than for the classical approach, at least 42.5% more SNPs were detected. The set of QTL-SNPs (peakQTL-SNPs) contains 3,034 SNPs (57 SNPs) for fat content and 3,593 SNPs (69 SNPs) for protein content. For the QTL approach, at least 12 times as many SNPs were declared important as in the reduced classical approach or metabolite approach. In contrast, in the peakQTL approach the number of SNPs is similar to the reduced classical approach and the metabolite approach. Finally in most cases, the average number of important SNPs was clearly smaller for important metabolites compared to milk traits, for the reduced classical approach (cf. Table 1).

In addition, we observed that the DGAT1-SNP was detected for all three investigated milk traits. Hence, it was evaluated how often the DGAT1-SNP was detected over all inner cross-validation sets for important metabolites. The DGAT1-SNP was identified for the following metabolites in all : arabitol, aspartic acid, and pyroglutamic acid, for fat and protein. Additionally, the DGAT1-SNP had an impact on 2-amino-butanoic acid and asparagine when studying protein. For pH value, the DGAT1-SNP was identified nine times based on the metabolite glycine.

### Enrichment analysis of important SNP subsets with respect to known QTL

For fat content and protein content, it was investigated if sets of metabolite SNPs or reduced SNPs were enriched in the set of QTL-SNPs for all 10 cross-validation sets. In Table 2, the observed 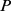-values as well as the number of expected and observed important SNPs located in QTL-SNPs are listed. For both investigated milk traits, the observed 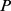-values were not significant on the significance level 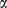 = 0.05, except in one case for the reduced classical approach (i.e., in the range of 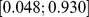 for fat). For the metabolite approach, however, the observed 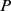-values were small and in almost all cases significant (e.g., in a range of 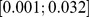 for fat). The important SNP positions for each milk trait which were detected in more than seven cross-validation runs with the metabolite approach are listed in Table S2. Also, it is marked if a SNP position lies in a known QTL. The SNP positions were specified, and aside from important SNPs in known QTL, further SNP positions were often detected, indicating their importance for the investigated milk trait.

**Table 2:** *P*-values resulting from rating the important SNPs for the reduced classical approach and the metabolite approach for each outer training set using an over-representation analysis.

	Reduced classical approach			Metabolite approach		
Trait	*P*-value	Expected	Observed	*P*-value	Expected	Observed
Fat content	0.737	2.56	2	0.010*	7.75	15
	0.588	3.01	3	0.001*	7.30	17
	0.930	2.56	1	0.032*	8.13	14
	0.048*	2.03	5	0.001*	9.26	20
	0.897	2.18	1	0.005*	7.90	16
	0.395	2.26	3	0.003*	9.56	19
	0.904	2.26	1	0.006*	9.48	18
	0.395	2.26	3	0.014*	6.62	13
	0.613	2.03	2	0.008*	8.28	16
	0.807	1.58	1	0.001*	6.77	16
Protein content	0.270	7.04	9	0.002*	20.76	35
	0.400	8.91	10	0.073	20.14	27
	0.488	8.56	9	0.017*	23.35	34
	0.921	6.86	4	0.011*	24.24	36
	0.815	7.93	6	0.025*	18.98	28
	0.717	7.04	6	0.004*	27.36	42
	0.690	7.93	7	0.069	28.79	37
	0.268	7.93	10	0.025*	23.97	34
	0.307	7.31	9	0.013*	20.41	31
	0.688	9.00	8	0.050*	18.54	26

The values of “Expected” and “Observed” correspond to the number of expected and observed important SNPs located in the belonging QTL.

* significance level 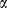 = 0.05.

### Predicting traits using different SNP subsets

**Figure 2:**
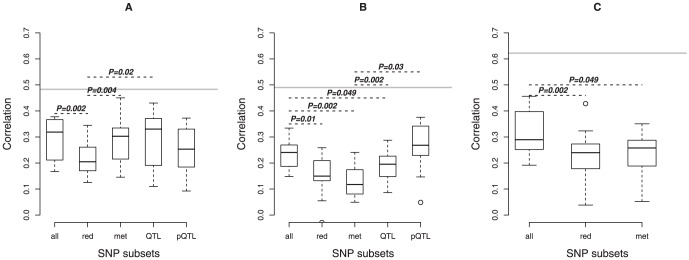
Boxplots of the observed precision of genetic value prediction over the 10 outer cross-validation runs for the classical approach (all), reduced classical approach (red), metabolite approach (met), QTL approach (QTL) and peakQTL-approach (pQTL). The following milk traits were investigated: fat content (A), protein content (B) and pH value (C). If two approaches differ significantly (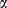 = 0.05), this is marked with a black dashed line and the observed P-value is given. The gray line represents a possible upper bound for the accuracy of prediction given as the square root of the estimated narrow-sense heritability based on a sire model.

For all investigated milk traits, boxplots of the observed prediction precisions for each SNP subset approach are presented in Figure 2. A significant difference between two approaches regarding the observed prediction precisions is marked with a black dashed line (*ρ* = 0.05); the corresponding observed P-value is also given. For fat content (Figure 2A), the reduced classical approach (*ρ* = 0.221) was surpassed by the following three approaches the classical approach (*ρ* = 0.299), the metabolite approach (*ρ* = 0.290) and the QTL approach (*ρ* = 0.293 for QTL-SNPs). Between these approaches no significant difference was observed. In addition, no significant difference was observed between these and the peakQTL approach (*ρ* = 0.254). Also no significant difference was observed between the reduced classical approach and peakQTL approach. Further, the highest single prediction precision of _ = 0.450 was observed for the metabolite approach, whereas for the classical approach the highest prediction precision was 0.377 and *ρ* = 0.430 for the QTL approach. In case of fat content, the most relevant approaches are the classical approach, the metabolite approach and the QTL approach, whereby compared to the classical approach and the QTL approach, less than 1% of the total amount of (40,317) SNPs were used for the prediction via the metabolite approach.

For protein content (Figure 2B), the classical approach (*ρ* = 0.237) outperformed the following three approaches: reduced classical approach, metabolite approach and QTL approach in terms of prediction precision. The peakQTL approach shows the highest mean prediction precision (*ρ* = 0.259), signi_cantly outperforming the metabolite approach. The highest single prediction precision of *ρ* = 0.376 was observed for the peakQTL approach. Moreover, no significant difference between the reduced classical approach (*ρ* = 0.147) and the metabolite approach (*ρ* = 0.126) or QTL approach (*ρ* = 0.188) was observed. For pH value (Figure 2C), the observed P-value for the comparison of the classical approach and the metabolite approach is 0.049 which is close to the bound of *ρ* = 0.05, whereas between the classical approach and the reduced classical approach a clearly significant difference was observed (P-value = 0.002). To validate that mean prediction precisions observed for metabolite SNP subsets and reduced SNP subsets were significantly different compared to those of random subsets, we implemented a resampling analysis. For fat content, a significant difference regarding prediction precision was observed for both approaches, whereas no significant difference occurred for protein content. For pH value, the observed resampling P-value was 0.051 for the metabolite approach and 0.055 for the reduced classical approach.

Additionally, the prediction ability of important milk metabolites was investigated using all SNPs and selected SNPs by SVS. The corresponding mean prediction precisions as well as the estimated h2 are listed in Table 1. The estimated h2 lie in a range of [0; 21] for fat content, [0:01; 0:48] for pH-value and [0:02; 0:41] for protein content. Most of the important milk metabolites (30 in total) have a small estimated h2 (23 in total), whereas only few have medium or high heritability. The observed prediction precisions for both SNP-subset (all SNPs and selected SNPs) were also rated. Rating was realized if the important metabolite was detected more than two times for the 10 cross-validations performed (22 milk metabolites in total). In 64% of all cases no significant difference between all SNPs and selected SNPs was observed. In general the mean prediction precisions were mostly similar although the estimated *h* ^2^ were different.

## Discussion

The aim of this study was to investigate the usefulness of metabolite profiles as additional information. For this purpose, three traditional milk traits were investigated. To predict these milk traits, a stochastic variable selection method was applied to different approaches resulting in various SNP subsets. The metabolite approach, representing our new proposed strategy, was realized by including information about metabolite profiles to select informative SNP subsets for the genotype to phenotype prediction step.

We could show that the observed mean precision of genetic value prediction using important SNPs of the metabolite approach was mainly more similar to the classical approach than to the reduced classical approach. Hence, our metabolite approach performed similarly but worked with a much smaller SNP subset compared to the classical approach. Further, significantly more important SNPs in known QTL regions were detected using the metabolite approach compared to the reduced classical approach. In the following we discuss our findings with emphasis on consequences and importance for future applications.

### Relevance of results

#### Milk trait prediction from milk metabolite profiles

In Step 1 of the “Analysis design”, where milk metabolites are used to predict milk traits to enable the detection of milk metabolites which are important for the investigated milk trait. For this step all 190 milk metabolites were used, which represents about 10% of the expected metabolites in cow's milk [36]. These milk metabolites were measured via GC-MS and thus cover predominantly short-chain water-soluble metabolites of the energy metabolism [6]. Moreover, milk metabolites mirror only a partial picture of the metabolic pathways and metabolites important for milk trait, since many of the latter are not contained in milk. In this context, “another aspect to be considered is that many of the measured milk metabolites are used for synthesis of milk components by the alveolar epithelial cells or are involved in intracellular metabolism, which leads to the questions of why they are measured in milk” [6].

In general, the recorded experimental measurements for milk metabolites and milk traits of the Holstein cows represent a snapshot of the current metabolic state and lactation stage, including the energy status which is known to change over the lactation stage. In our data, more important milk metabolites were detected for protein content than for fat content and pH value (cf. Table 1). Also the mean prediction precision over the inner 10 cross-validation runs were clearly higher for protein content compared to the other both milk traits. This leads to the assumption that the measured part of the milk metabolome includes more relevant metabolites for protein content. The detected milk metabolites can be seen as possible biomarker candidates, although no multiple measurements of milk traits as well as milk metabolites were available [6]. In Melzer et al. [6] these measurements were intensively studied and some of the important milk metabolites could be assigned a possible role with respect to the investigated milk trait. As an example, spermidine was detected as important for protein content. It is known that this metabolite is involved in the production of protein [37]–[39]. In Melzer et al. [6] it was also mentioned that their physiological role is necessary to further investigations as well as to study the degree of change of the milk metabolites over the course of lactation stage. In this context it would be advantageous to investigate also correlation structure of metabolite profiles to find possible relations or functional grouping structures. Hence, such structures could be used related to a priori functional knowledge like for example metabolite pathways. Such knowlegde could help to understand the functional basis of biosignature candidates as well as to improve learning algorithms which are able to use a priori information (e.g., [40]). Finally, the important milk metabolites which were revealed should be validated with another data set.

#### Comparison between the reduced classical approach and metabolite approach

The presented metabolite approach allows for the selection of important SNPs regarding an investigated milk trait, and it represents a new strategy compared to proposed SNP subset selection strategies found in the recent literature, e.g., Habier *et al.* [11] and Moser *et al.* [13]. The metabolite approach enables investigations in two different directions. On the one hand, using metabolite profiles to predict milk traits enables detecting important metabolites for an investigated milk trait. On the other hand, being the focus of the current study, the important metabolites were used to determine associated SNPs (Step 2 “Analysis design”) which were involved in milk trait prediction (Step 3 “Analysis design”). Both steps were also performed for the reduced classical approach. Our findings regarding both approaches are discussed in more detail in the following, especially in order to find evidence that SNPs detected by the metabolite approach are important for the investigated milk traits, and to show that the SNP subsets, e.g., detected for fat content, can have a clear advantage for milk trait prediction compared to the reduced classical approach:

- Importance of selected SNPs by SVS for investigated milk trait: Significantly more SNPs located in known QTL were detected using the metabolite approach compared to the reduced classical approach (Table 2) for fat content and protein content, which was surprising. This finding comes with the restriction that not all QTL for a trait are known. A possible reason could be that for the reduced classical approach, despite a possibly large number of important SNPs, some of the genetic effects of SNPs are overlaid and may cancel out (as discussed in [41]), if the complex milk trait itself is investigated. It is imaginable that important SNPs for single milk metabolites show stronger genetic effect sizes. To enable a clear statement, further investigations are necessary regarding testing each or groups of SNPs detected for an important metabolite as well as groups of important metabolites for their relevance for the milk trait.
- A SNP with an important impact on a milk trait may also have an impact on at least one metabolite: The DGAT1-SNP had an impact on at least one metabolite important for protein and fat. The DGAT1-SNP was also detected using the reduced classical approach for both milk traits. The DGAT1-SNP coincided with the SNP position with the largest importance found by Cole *et al.* [42] or Weller *et al.* [14]. For pH value, the DGAT1-SNP was observed 10 times via the reduced classical approach, and nine times for the metabolite glycine via the metabolite approach (Table 1). In general, it is not surprising that this SNP position was not detected in all cases, due to the smaller number of the important metabolites for this milk trait. This observation supports our expectation that a SNP with a significant genetic effect on a milk trait also shows a significant effect on at least one of the important metabolites.

To which extent it hold true for other significant detected SNPs is not clear. Since known genome loci with an high impact on a milk trait are rare. To our knowledge only the two QTNs DGAT1 and ABCG2 are known [14]. In our case, before this study it was not known that the DGAT1-SNP will also be detected for milk metabolites which are associated with the investigated milk trait. This could be seen as a first indication that SNPs detected for milk metabolites could be also important for the investigated milk trait.

- Important SNP subsets in respect to milk trait prediction: For fat content, the mean prediction precision was significantly higher for the metabolite approach (*ρ* = 0.290) than for the reduced classical approach (*ρ* = 0.221). In this case, no significant difference was observed between the classical approach and the metabolite approach. For pH value, the difference regarding the observed prediction precisions between the classical approach and the metabolite approach was very small. In this context, it is expected that no significant difference will be observed, if e.g., a more suitable part of the metabolome is measured for pH value. For protein content it seems difficult to find a suitable SNP subset to obtain an appropriate prediction precision without a priori information (see below). The reason for this result might be the genetic architecture of this milk trait, since protein content probably depends on many QTL with small genetic effect sizes, which makes it hard to detect the relevant SNPs for this trait. This assumption is supported by the finding of the resampling analysis, which yielded no significant difference between the reduced classical approach and the metabolite approach with regard to prediction precision. In this case, we assume that milk metabolites have a similar complex underlying genetic architecture as milk traits. Another explanation for the bad results of the metabolite approach in the case of protein content could be that we did not measure the relevant milk metabolites for this trait.

In general, the resampling analysis also con_rms indirectly that the detected important SNP subsets for the metabolite approach and the reduced classical approach are important for fat content and pH value. Findings for fat content and pH value indicate that the SNP subsets selected by the metabolite approach are more suitable for prediction of the investigated milk traits than using the reduced classical approach. As mentioned above the metabolite approach detected a significant number of important SNPs located in known QTL, showing the relevance of most of the important SNPs for investigated milk traits. This suggests that the other important SNPs could be relevant or are possibly located in unknown QTL for the investigated milk traits. To enable a clear statement further investigations are necessary. Important SNPs observed with the metabolite approach in at least eight cross-validation runs are listed in Table S2.

- Finally, it is expected that more than 2,000 metabolites exist in cow's milk (as mentioned above). In our case, we analyzed about 10% of them, originating primarily from the central carbon and energy metabolism. We suppose that the prediction precision will increase if more relevant metabolites are measured for the investigated milk trait, which will become possible in the near future as GC-MS databases increase.

#### Using SNP subsets based on a priori information in respect to their role for the investigated milk trait

For protein and fat, the prediction precisions obtained via the QTL approach are also presented in Figure 2. In general, the QTL approach has two disadvantages:

- First, not all QTL for a trait are known.
- Second, most of the QTL regions comprise a long segment of the corresponding chromosome (Table S1). Some of the selected SNPs in these regions are not necessarily important for the investigated milk trait, because most QTL regions have a QTL peak location, which is the position with the highest or lowest value depending on the used test statistic.

Hence, it was further investigated if a higher prediction precision is observed if only the peak locus is considered instead of the whole QTL region. The results show that similar prediction precisions were obtained with the peakQTL approach compared to the QTL approach. Moreover, we observed that peakQTL approach showing larger prediction precision than the QTL approach in case of protein content. A significant difference between the classical and the QTL approach was observed, but not between the classical approach and peakQTL approach. Further, the resampling results obtained for the reduced classical approach (88 SNPs instead of 69 SNPs peakQTL approach) show that the observed prediction precision is significant. The same holds true for fat content. In this context, it is to recommended to use SNPs which are close to peaks of QTL instead of the QTL approach, but it is not clear to which extent possible additional SNPs are included in these QTL regions which have also a relevance for the investigated milk trait. Hence, further investigations are necessary and such investigations could be also relevant to improve variable selection methods such as SVS.

### Benefits and constraints of methods

In general, as mentioned above, it would be desirable to have multiple measurements from milk metabolites and milk traits.

To determine important metabolites for an investigated milk trait the regression methods and were applied. Both methods were selected based on a preliminary study in which the same settings were used and reliable results were obtained [6].

The important SNP subsets for the metabolite approach and the reduced classical approach were analyzed in various tests to determine their importance for the investigated milk trait more precisely. For this, other data in the form of known QTL were used to enable the confirmation of some of the important SNPs. A resampling approach was realized to quantify the significance of the observed prediction ability for the metabolite approach as well as for the reduced classical approach.

In the recent literature (e.g., [23], [24]), it is often mentioned, that traits with a low heritability require a larger sample size than traits with moderate or high heritability to obtain an acceptable prediction precision. This implies that more false-positive SNPs would be found for traits with low heritability if the sample size is too small., to test if an appropriate prediction precision can be obtained for each metabolite using the resampling approach as above. On the one hand, this would allow for a deeper insight into the genetic architecture of a metabolite. On the other hand, such information could be used to improve our approach. Our findings show that, even if the heritability of the metabolite was not taken into account, an appropriate mean prediction precision was for fat (e.g., 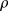 = 0.29 and 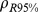 = 0.23 for the metabolite approach) but not for protein (e.g., 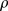 = 0.13 and 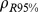 = 0.21 for the metabolite approach).

In this study, each analysis step was evaluated separately, and based on the observed result the next step was realized. We suppose, that an embedded approach, optimizing our three step approach in a common cross-validation design could be superior. Also, conceivable alternatives would be to use other data from the metabolome or genome level to optimize filter criteria or to use such information for weighting SNPs.

In addition, in this study the pedigree information was neglected based on following reasons:

- The prediction of genetic values was realized without considering the pedigree information, since marker data and phenotypes are adequate and sufficient for the genetic value prediction [43].
- To estimate the heritabilities is most common to use an animal model [24], since the animal model is more accurate in some cases than the sire model. The advantage of the sire model is that it needs clearly less equations [44], i.e., the covariance matrix does not need to be created, and thus can be estimated without much effort. In addition, the narrow-sense heritability of milk traits was also estimated based on an animal model by Dr. D. Wittenburg, including the pedigree information. The estimated heritabilities of the animal model as well as of the sire model were similar (results not shown).
- In our case, the sire model was suitable, since heritability was mainly estimated to obtain an upper bound for the prediction precision of the genetic value for the investigated trait. Also it enables a better correction for the fixed effects for the investigations of relationships between milk metabolites and milk traits.

In this context, it should be investigated if the prediction of milk traits from metabolite profiles can be improved if additionally the pedigree information are considered.

Finally, it is recommended to evaluate our approach for the inclusion of non-additive effects. For example, Lee *et al.* [45] and Toro & Varona [46] have shown that using an additive-dominance model results in a larger prediction precision.

## Conclusions

In our study, we demonstrated the usefulness of considering metabolite profiles for the genetic value prediction realized in a three step approach based on different SNP subsets. Our results show that using the metabolite approach led to a prediction precision similar to that of the classical approach, but required less than 1% of the total amount of (40,317) SNPs. In most cases, the approach performed better than the reduced classical approach. Our approach allows for a deeper insight into the associations between the different levels of the genotype-phenotype map (genotype-metabolome-phenotype) and to analyze the reported important and their associated SNPs regarding their role for the investigated milk trait. It was possible to prove the importance of some of the observed important SNPs via the metabolite approach using known QTL positions. The here presented investigations come with the restriction that for all used measurements, i.e., milk metabolites and milk traits, only a single measurement was available.

The success of our approach depends, among other things, on the underlying genetic architecture of the investigated milk trait, and presumably on the measured part of the milk metabolome.

## Supporting Information

Figure S1 **Correlations between important milk metabolites for all investigated milk traits.** The correlation values were adapted from [6].
(PDF)

Table S1 **Known QTL regions or QTL peaks, which were filtered from the cattleQTL database** [**25**] **.** The following criteria were applied: trait name equal to milk fat percentage and milk protein percentage, analysis type equal to QTL, breed equal to Holstein, and chromosome number and both flanking markers or peak markers had to be available. Based on the marker names it was possible to locate the marker in the physical unit base pair (bp) using the annotation Btau4.2 of the bovine genome from the National Center for Biotechnology Information (NCBI; [26]). Also, the known QTN DGAT1 [15] was considered QTL for both milk traits. The SNP marker ARS-BFGL-NGS-4939 was located directly in the DGAT1 region and was used in the analysis.
(TEX)

Table S2 **Important SNP markers, occurring in more than seven cross-validation (CV) runs, for each milk trait obtained via the metabolite approach.** For this analysis, the 10 training sets from the outer cross-validation was used.
(PDF)
