# Supplementary material for: Integrating Milk Metabolite Profile Information for the Prediction of Traditional Milk Traits Based on SNP Information for Holstein Cows
Source: PLoS One. 2013 Aug 21;8(8):e70256. doi: 10.1371/journal.pone.0070256 (PMC3749218; doi:10.1371/journal.pone.0070256)
Supplement: Figure S1 — Correlations between important milk metabolites for all investigated milk traits. The correlation values were adapted from [6]. (PDF) [file pone.0070256.s001.pdf]

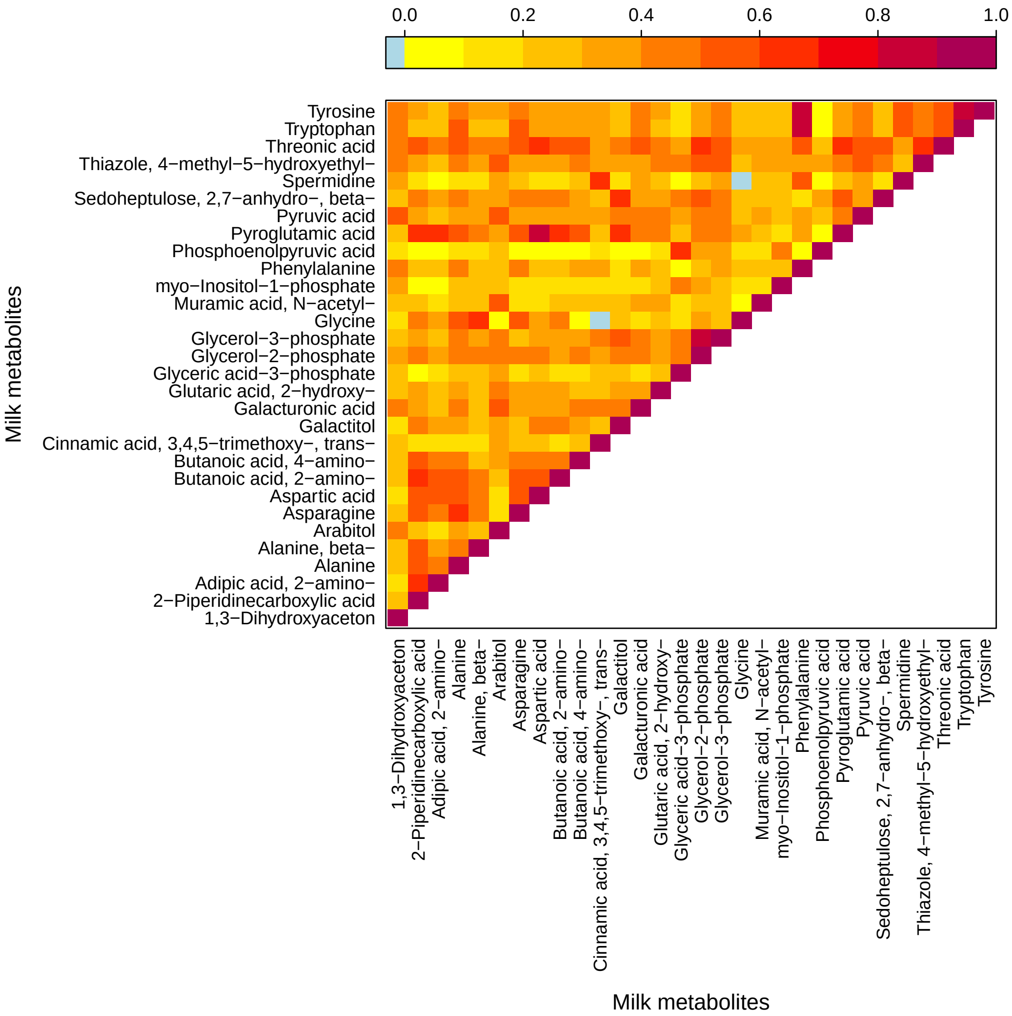

Figure S1. Correlations between important milk metabolites for all investigated milk traits. The correlation values were adapted from [1].

References

1. Melzer N, Wittenburg D, Hartwig S, Jakubowski S, Kesting U, et al. (2013) Investigating associations between milk metabolite profiles and milk traits of Holstein cows. J Dairy Sci 96: 1521 - 1534.
