# Supplementary material for: Integrating Milk Metabolite Profile Information for the Prediction of Traditional Milk Traits Based on SNP Information for Holstein Cows
Source: PLoS One. 2013 Aug 21;8(8):e70256. doi: 10.1371/journal.pone.0070256 (PMC3749218; doi:10.1371/journal.pone.0070256)
Supplement: Table S2 — Important SNP markers, occurring in more than seven cross-validation (CV) runs, for each milk trait obtained via the metabolite approach. For this analysis, the 10 training sets from the outer cross-validation was used. (PDF) [file pone.0070256.s003.pdf]

Table S2. Important SNP markers, occurring in more than seven cross-validation (CV) runs, for each milk trait obtained via the metabolite approach. For this analysis, the 10 training sets from the outer cross-validation was used.

SNP marker	Chromosome	Position in basepair	QTL region	QTL peak	Count in 10 CV runs
**Fat content**					
Hapmap42518-BTA-34464	2	11,5611,887			8
Hapmap50895-BTA-122111	4	24,553,482			10
ARS-BFGL-NGS-64882	7	81,392,639			8
ARS-BFGL-NGS-17358	8	102,312,736			9
BTA-63354-no-rs	10	33,150,420			9
Hapmap58072-rs29010006	12	63,149,779			8
BTB-01123944	13	6,281,252			8
ARS-BFGL-BAC-11928	13	29,191,010			9
ARS-BFGL-NGS-57820	14	236,532			8
ARS-BFGL-NGS-4939	14	443,937		✓	10
ARS-BFGL-NGS-107379	14	679,600			10
BFGL-NGS-113453	14	30,002,363	✓		9
Hapmap48989-BTA-101611	14	34,879,141			9
BTB-01157350	17	1,384,889			9
BTB-01951543	20	49,918,496			8
Hapmap39714-BTA-111678	21	18,811,723			8
ARS-BFGL-NGS-69616	21	22,250,027			9
**Protein content**					
Hapmap39813-BTA-21834	1	53,621,500			8
BTB-01978832	2	135,640,997			8
Hapmap42708-BTA-86534	3	50,850,297	✓		9
Hapmap50895-BTA-122111	4	24,553,482			10
BTB-00234759	5	94,104,074			8
BTB-01534149	6	66,230,967	✓		9
ARS-BFGL-NGS-29273	7	5,652,920			8
BTA-87610-no-rs	7	57,673,607			9
ARS-BFGL-NGS-64882	7	81,392,639			10
Hapmap49034-BTA-115720	8	88,958,729			8
Hapmap39516-BTA-82096	8	90,551,290			8
ARS-BFGL-NGS-17358	8	102,312,736			9
ARS-BFGL-NGS-100341	9	52,911,776	✓		8
BTA-63354-no-rs	10	33,150,420			9
BTB-01972463	11	16,078,669			9
BTA-93103-no-rs	11	33,330,852			8
ARS-BFGL-NGS-32722	11	95,942,521			10
Hapmap58072-rs29010006	12	63,149,779			9
BTB-01123944	13	6,281,252			8
ARS-BFGL-BAC-11928	13	29,191,010			8
BTA-32552-no-rs	13	42,633,511			9
ARS-BFGL-NGS-38064	13	54,700,987			8
ARS-BFGL-NGS-104967	13	55,847,196			9
ARS-BFGL-NGS-5166	13	56,045,155			9
ARS-BFGL-NGS-57820	14	236,532			10
ARS-BFGL-NGS-4939	14	443,937		✓	10
ARS-BFGL-NGS-107379	14	679,600			10
Hapmap30086-BTC-002066	14	1,490,178			9
BFGL-NGS-113453	14	30,002,363			9
Hapmap41433-BTA-114994	14	33,550,219			9

SNP marker	Chromosome	Position in basepair	QTL region	QTL peak	No. of detection in 10 CV runs
Hapmap48989-BTA-101611	14	34,879,141	✓		9
BTB-00642563	16	43,274,808			9
BTA-40059-no-rs	16	694,769,20			9
BTB-01157350	17	1,384,889			9
Hapmap49611-BTA-44077	17	26,304,955			8
Hapmap42359-BTA-90829	18	20,737,022			9
Hapmap49176-BTA-43744	18	49,761,247			10
ARS-BFGL-NGS-34276	18	49,839,669			9
Hapmap34814-BES8_Contig361_961	19	20,361,224			8
ARS-BFGL-NGS-11174	19	43,331,499			8
ARS-BFGL-NGS-69616	21	22,250,027			9
Hapmap49032-BTA-115439	22	54,473,771			9
BTA-54892-no-rs	22	54,503,230			8
BTA-112061-no-rs	23	38,207,532			8
ARS-BFGL-NGS-22050	25	27,843,968			10
ARS-BFGL-BAC-42500	25	28,002,712			10
ARS-BFGL-NGS-41056	26	20,364,191			9
BTB-00624015	27	20,648,605			10
ARS-BFGL-NGS-18177	29	4,598,272			10
ARS-BFGL-NGS-20615	29	5,294,603			8
**pH value**					
ARS-BFGL-NGS-103495	8	9,605,960			9
BTA-63354-no-rs	10	33,150,420			10
BTA-98790-no-rs	13	25,929,330			9
ARS-BFGL-BAC-12549	13	54,601,927			9
ARS-BFGL-NGS-57820	14	236,532			8
ARS-BFGL-NGS-4939	14	443,937			9
ARS-BFGL-NGS-107379	14	679,600			10
ARS-BFGL-NGS-107810	15	66,121,820			8
ARS-BFGL-NGS-40131	17	10,374,164			8
Hapmap49611-BTA-44077	17	26,304,955			9
Hapmap49176-BTA-43744	18	49,761,247			10
BTA-23545-no-rs	18	50,063,553			8
ARS-BFGL-NGS-22050	25	27,843,968			10
ARS-BFGL-BAC-42500	25	28,002,712			10
ARS-BFGL-NGS-100347	25	28,535,691			9
BTB-00624015	27	20,648,605			10
Hapmap42281-BTA-63982	28	29,215,768			8
ARS-BFGL-NGS-18177	29	4,598,272			8

✓- SNP is located in a QTL
